# Potential new applications of immunotherapy for neuroendocrine neoplasms: immune landscape, current status and future perspectives

**DOI:** 10.20892/j.issn.2095-3941.2022.0489

**Published:** 2022-10-18

**Authors:** Rilan Bai, Wenqian Li, Jiuwei Cui

**Affiliations:** 1 Cancer Center, the First Hospital of Jilin University, Changchun 130021, China

**Keywords:** Neuroendocrine neoplasms, immunotherapy, predictive biomarker, hormones

## Abstract

Neuroendocrine neoplasms (NENs) are a highly heterogeneous class of tumors arising from neuroendocrine cells and peptidergic neurons. After failure of first-line treatment, patients have poor prognosis and limited treatment options. Immune checkpoint inhibitors (ICIs) may be a powerful means of increasing therapeutic efficacy for such patients, but ICIs alone have low response rates and short disease control durations in most NENs and may be effective for only a portion of the population. ICIs combined with other immunotherapies, targeted therapies, or cytotoxic drugs have achieved some efficacy in patients with NENs and are worthy of further exploration to assess their benefits to the population. In addition, accumulating experimental and clinical evidence supports that the interaction between neuroendocrine and immune systems is essential to maintain homeostasis, and assessment of this broad neuroendocrine-immune correlation is essential for NEN treatment. In this review, we summarize the immune microenvironment characteristics, advances in immunotherapy, predictive biomarkers of ICI efficacy for NENs, and the effects of common endocrine hormones on the immune system, highlighting possible new application areas for this promising treatment in neglected NENs.

## Introduction

Neuroendocrine neoplasms (NENs) are a highly heterogeneous class of tumors arising from neuroendocrine cells and peptidergic neurons, which express neuroendocrine markers and produce bioactive amines and/or polypeptide hormones. NENs are classified as well-differentiated neuroendocrine tumors (NETs) and aggressive, poorly differentiated neuroendocrine carcinomas (NECs)^1,2^. NENs may appear in different parts of the body, most commonly in the gastroenteropancreatic compartment (up to 70% of cases) and respiratory tract (approximately 20% of cases)^3–5^, whereas those arising from other regions, such as the genitourinary tract, the female reproductive system, and Merkel cells of the skin are less common. Their behavior, metastatic potential, and prognosis are highly variable, depending on the site of origin, differentiation grade, and proliferation index^4^. Treatment approaches for NENs vary widely and are based on the location, grade, and stage of the primary lesion^6^. The cornerstone treatments for well-differentiated NETs are surgery, local ablation therapy, antisecretory and anti-proliferative drugs, such as somatostatin receptor ligands and peptide receptor radionuclide therapy, and targeted therapies; however, their rates of effectiveness are low^7^. High-grade NECs are biologically similar to small cell lung cancers (SCLCs), and are characterized by rapid disease progression and high sensitivity to platinum-based chemotherapy, yet no standard regimen exists for NECs after second-line treatment, and patients who fail first-line treatment have very poor prognosis. The reported overall survival (mOS) of patients with poorly differentiated NECs is 11 months, and the progression-free survival (mPFS) is 4 months^8,9^. Limited drugs are available for advanced NENs, and effective treatments remain lacking.

Immunotherapy is the most important breakthrough in the field of cancer therapy in recent years, and immune checkpoint inhibitors (ICIs) have made major breakthroughs in cancer therapy and have been approved for the treatment of various types of cancer^10,11^. For NENs, immunotherapy is used primarily to treat lung and skin tumors, and ICIs are approved for SCLCs and Merkel cell carcinomas (MCCs)^12–15^, because both types have high tumor mutational burden (TMB) and environmental causes of immunogenicity^16^. For other NENs, several early trials and clinical studies have evaluated the efficacy of ICIs and provided preliminary insights into the roles of these therapies. Overall, the results of exploratory studies in NENs have shown that the efficacy of immunotherapy alone is limited but may be considered for portions of the population. ICIs combined with other immunotherapies, targeted therapy, or cytotoxic drugs have achieved some efficacy in patients with NENs and are worthy of further exploration for their benefits to the population. Herein, we summarize the immune microenvironment characteristics, advances in immunotherapy, predictive biomarkers of ICI efficacy for NENs, and the effects of common endocrine hormones on the immune system, highlighting possible new areas of application for this promising treatment in neglected NENs.

## Characteristics of the tumor immune microenvironment of neuroendocrine neoplasms

NETs usually exhibit an immunologically “cold” tumor immune microenvironment, owing to the lack of immunocompetent cellular components, low tumor antigens, and other factors^17,18^; in contrast, NECs may be a more suitable target for immunotherapy, given their extensive mutation load and denser immune infiltration^19^. In terms of PD-L1 expression, 8.99% of G1, 12.37% of G2, 37.04% of G3, and 48.91% of NECs had ≥ 25% positive PD-L1 membrane staining in tumor cells or tumor-infiltrating lymphocytes (TILs)^20^. Lamarca et al.^21^, in a similar series of 70 tissue samples from small bowel NETs (sb-NETs), have observed 2% Ki-67 positivity, and PD-L1 positivity (≥ 5% membrane expression) in 12.8% of tumor cells and 24.3% of TILs. PD-L1 expression is significantly associated with a higher WHO tumor grade^22,23^, and poorer PFS and OS in NENs^24–26^. A recent analysis of 102 NETs of different grades and primary sites has indicated that PD-L1 expression is highest and lowest in lung and ileal NETs, respectively, whereas PD-L2 expression is highest in pancreatic NETs^27^. In addition, depleted and regulated TILs are enriched in PD-L1-positive NETs but diminished in G3 well-differentiated NETs, thus suggesting that immune tolerance in NETs may be driven by PD-L1/2 expression, and NETs that express PD-L1 and with TILs might benefit from PD-L1 inhibition. However, other studies have found no association between PD-L1 expression and grade or prognosis^26,28^.

A recently published article analyzing the genomic landscape of late-stage NENs has measured TMB through whole genome sequencing and found a lower TMB for NENs (1.09 mut/Mb) than NECs (5.45 mut/Mb), and a higher number of indels, structural variants, and polyploid genomes in NECs, according to an analysis of the types of genomic alterations^29^. Similarly, in the well-differentiated pancreatic NET (pNET) cohort reported by Scarpa et al.^30^ (*n* = 98), the TMB was 0.82 mut/Mb. Moreover, in the grade 3 NET cohort reported by Venizelos et al.^31^ (*n* = 29), the TMB was 4.6 mut/Mb, and that in the gastro-entero-pancreatic (GEP)-NEC (*n* = 152) cohort was 5.1 mut/Mb. Notably, lung NETs have been shown to have higher overall TMB levels. A retrospective study by Chi et al.^32^ has reported a TMB of lung NETs of 11.0 mut/Mb, and a retrospective study by Sabari et al.^33^ has found a significantly higher TMB for large cell neuroendocrine carcinomas (LCNECs) than SCLCs (15.3 mut/Mb *vs*. 8.2 mut/Mb) and non-small cell lung cancers (NSCLCs) (15.3 mut/Mb *vs*. 5.7 mut/Mb). In addition, higher T cell infiltration in the immune microenvironment has been observed for highly malignant NETs/NECs. Through multiplex fluorescence immunohistochemistry for quantitative analysis, the number of TILs and PD-L1^+^ TILs has been found to be significantly greater in pNECs than pNETs, and PD-L1 high T-lymphocyte infiltration is significantly greater with increasing grade in pNETs^17^. Da Silva et al.^34^ have reported T cell immune infiltration, with high density T cell infiltration (CD4^+^, CD8^+^, and CD45RO^+^ cells) in the intratumoral compartment in 14%–48% of sb-NETs and 32%–65% of pNETs, and low levels of FOXP3^+^ regulatory T cells (Tregs) cells in both cohorts. Nevertheless, the immunological characteristics of NENs are not fully understood, and more knowledge regarding the complex immune landscape of these heterogeneous tumors must be obtained to clarify the therapeutic and prognostic value of these NEN characteristics.

## Effects of hormones secreted by the neuroendocrine system on the immune system

Functional NENs secrete a variety of hormones and cause a variety of neuroendocrine syndromes, thus affecting the tumor immune microenvironment and systemic immune status. A growing body of experimental and clinical evidence supports that the interaction between the neuroendocrine and immune systems is essential for the maintenance of homeostasis^35^. For example, hormones such as prolactin (PRL), growth hormone, cortisol, and sex hormones regulate the differentiation and function of immune system cells and cytokine production^36^, and vice versa. Assessing this broad neuroendocrine-immune correlation is essential for understanding NENs. Adrenocorticotropic hormone (ACTH) generally suppresses immune responses, but certain functions can be enhanced. For example, in an investigation of the effect of ACTH on cytotoxicity in T lymphocytes previously sensitized *in vivo*, ACTH showed no significant effect on primary mixed lymphocyte responses but enhanced secondary (memory) cytotoxic responses by as much as 100% after 2 days of treatment^37^. ACTH also inhibits concanavalin A-stimulated T lymphocyte mitosis. Mitotic inhibition is stronger in immature thymocytes than in mature thymocytes. Furthermore, the finding that IFN-γ is elevated in culture suggests that ACTH may enhance memory cytotoxic responses through multiple mechanisms such as direct cellular alterations or synergy with regulatory cytokines^37^. A sexual dimorphism exists in the expression of innate and adaptive immune responses^38^, as a result of the effects of androgens and estrogens on the immune system. Estrogen can promote or protect against autoimmune diseases^39^, and androgens have been described as suppressors of inflammation and immune function^40,41^, which directly promote neutrophil differentiation from myeloid progenitors, inhibit dendritic cell differentiation and function, and inhibit B-cell and T-cell lymphopoiesis, but may increase the risk of cancer development^42^. Notably, a robust indicator of response to immunotherapy is intratumoral expression of IFNG^43,44^, which is inhibited by androgens^45^. Inhibition of androgen receptor activity in CD8^+^ T cells has been found to prevent T cell exhaustion and increase responsiveness to PD-1 targeted therapy by significantly increasing cytokine production and IFN-γ expression in CD8^+^T cells^46^. PRL has immunomodulatory effects^47^, and its secretion is stimulated by cytokines such as IL-1 and IL-2, and inhibited by endothelin-3 and IFN-γ^48^. PRL increases IL-2 synthesis and secretion, and stimulates IFN-γ production by natural killer (NK) cells and lymphocytes; promotes maturation of thymic CD4^+^ T and CD8^+^ T lymphocytes, and stimulates immunoglobulin production by plasma cells; promotes the development of antigen-presenting cells expressing major histocompatibility class II molecules; and stimulates IL-1β production by macrophages^48^. In addition, a variety of other stimuli also regulate the body’s immunity through diverse mechanisms. For example, growth hormone promotes neutrophil differentiation; antibody and transcription factor synthesis; T cell proliferation, adhesion, and cytotoxic activity; and production of IL-1, IL-2, and IFN-γ^49^.

## Advances in ICIs for neuroendocrine neoplasms

**Figure 1:**
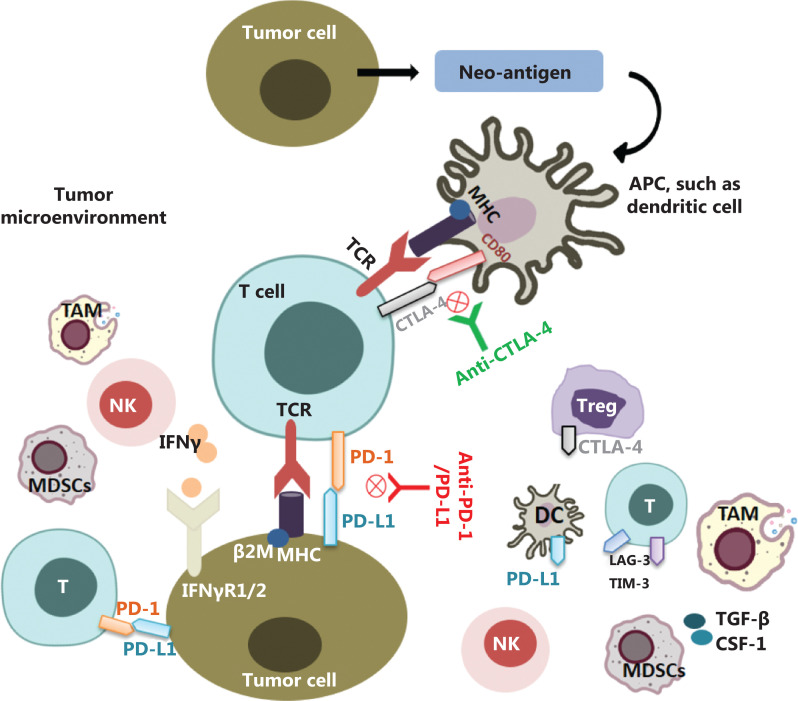
The immune response process of tumor cells and the main mechanism of action of ICIs. First, tumor cells release antigens that are taken up by antigen-presenting cells. These cells present tumor antigens to naive T cells and activate them. PD-1 in activated T cells interacts with PD-L1 in tumor cells, thereby suppressing immune responses. CD80 on antigen-presenting cells binds CTLA-4 on activated T cells and suppresses immune responses. ICIs, such as anti-PD-1/PD-L1 and anti-CTLA-4, block the interaction of immune checkpoint molecules between tumor cells and immune cells, or between immune cells and antigen presenting cells, thereby activating immune cells and improving anti-tumor immune responses.

The immune response process of tumor cells and the main mechanisms of action of ICIs are described in detail in **Figure 1**. However, evidence of the efficacy and safety of ICIs in the treatment of NENs remains limited; phase I/II trials have evaluated the roles of ICIs and combinations in the treatment of NENs^50–55^, but randomized controlled phase III trials have not been conducted. A recent systematic review and meta-analysis of 636 patients with NENs treated with ICIs has reported an objective response rate (ORR) of 10%, an overall disease control rate (DCR) of 42%, an mPFS of 4.1 months, and an mOS of 11 months, thereby demonstrating the overall effectiveness of ICIs in the treatment of patients with NENs^56^. Park et al.^57^ have systematically evaluated the effectiveness of ICIs in patients with advanced or metastatic NENs. In a pooled analysis of 10 studies with 464 patients, the overall ORR was 15.5%, but the values varied by primary site (thoracic, 24.7%; gastroentero-pancreatic, 9.5%), tumor differentiation (poorly differentiated, 22.7%; well differentiated, 10.4%), and drug regimen (combined therapy, 25.3%; monotherapy, 10.1%). For patient-tailored management, changes in ICI treatment efficacy according to tumor differentiation and drug regimen should be considered. The promising efficacy and favorable safety profiles of ICIs indicate an opportunity to expand the therapeutic promise for NENs. A summary of the main trial results and ongoing studies of immunotherapy and combination therapy is provided below.

### Advances in ICI monotherapy for neuroendocrine neoplasms

The effectiveness of ICI monotherapy for NENs is limited, particularly for poorly differentiated tumors^58,59^. Pembrolizumab is the most widely studied immunotherapeutic agent for NENs. The phase Ib Keynote-028 study showed that 16 patients with PD-LI-positive pNECs had an ORR of 6%, and 12-month PFS and OS rates of 27% and 87%, respectively; 25 cohorts of patients with typical carcinoid (TC) or atypical carcinoid (AC) had an ORR of 12%, and 12-month PFS and OS rates of 27% and 65%, respectively^50,60^. The phase II basket trial Keynote-158 study has reported that, for well-differentiated NETs that failed standard treatment, pembrolizumab treatment had an ORR of only 3.7%, an mPFS of 4.1 months, a 6-month PFS of 39.3%, and an mOS of 24.2 months, with good safety data^51^. Although efficacy is limited in patients with NECs overall, pembrolizumab has been approved by the U.S. Food and Drug Administration (FDA) for the treatment of SCLCs in patients with evidence of disease progression during or after platinum-based chemotherapy and at least one other prior therapy, on the basis of on pooled data from the SCLC cohorts in the Keynote-158 and Keynote-028 trials. In addition, the results of a prospective randomized phase II trial evaluating pembrolizumab in 19 patients with NECs and 9 patients with G3 NETs have shown no response^16^. In a trial of 14 patients with extrapulmonary poorly differentiated NECs, the ORR of pembrolizumab treatment was 7%, and only one patient achieved complete response^58^. In the phase II KEYNOTE-017 trial, the ORR of pembrolizumab in first-line treatment of advanced MCCs was 56% (59% for virus-positive and 53% for virus-negative patients, regardless of PD-L1 expression), the 24-month PFS was 48.3%, the mPFS was 16.8 months, the 24-month OS was 68.7%, mOS was not reached, 28% had grade 3–4 treatment-associated adverse events (TRAEs), 7 patients (14%) discontinued pembrolizumab because of AEs, and 1 patient died from treatment^61^. Avelumab is the only PD-L1 inhibitor used as a single agent in prospective clinical trials in GEP-NENs, and 3 phase II clinical trials (NCT03278405^62^, NCT03278379^63^, and NCT03147404) have been conducted to evaluate avelumab in patients with G2/3 NETs or NECs. However, no patients achieved an objective response to avelumab treatment. The phase II AVANEC trial assessed the activity of avelumab in 29 patients with high-grade NENs of different origins, with an ORR of 6.9% and mPFS of 16 weeks^64^. In a retrospective study conducted at the Mayo Clinic, 3 patients with G3 NETs showed no objective response to ICIs^65^. In the JAVELIN Merkel 200 trial, the ORR of avelumab therapy in a cohort of patients with chemotherapy-refractory MCCs was 33.0%, 74% had a sustained response for more than 1 year, and the treatment was well tolerated^62,66^; interim results in the first-line MCC cohort with avelumab showed an ORR of 62.1%, an estimated DOR (at least 6 months) of 83%, and no grade 4 or 5 adverse events^67^. Walker et al.^68^ confirmed the positive results observed in the JAVELIN trial, in a study of nearly 500 patients with MCCs or progressive MCC. On the basis of these results, the FDA (avelumab and pembrolizumab) and the European Medicines Agency (avelumab) have approved ICIs as first-line or subsequent treatments for MCC. In the ongoing phase I/II CheckMate-358 study (NCT02488759, testing nivolumab treatment in participants with virus-positive and virus-negative solid tumors), the preliminary data for 25 patients with MCCs have indicated an ORR of 68%. The 3-month PFS and OS rates were 82% and 92%, respectively; 20% of patients had grade 3–4 TRAEs, and 12% discontinued treatment because of toxicity^69^.

Spartalizumab is a novel high-affinity humanized anti-PD-1 antibody that blocks the binding of PD-L1/L2 to PD-1^52^. A phase II trial has evaluated spartalizumab in 4 cohorts: well-differentiated (WD) GI-NETs (*n* = 32), WD pNETs (*n* = 33), WD thoracic NETs (*n* = 30), and GEP-NECs (*n* = 21), with ORRs of 0%, 3%, 20%, and 4.8%, respectively ^70^. Interestingly, patients with higher PD-L1 expression or more CD8^+^ cell infiltration at baseline evaluation showed higher ORR^70^. In a phase II, single-arm, open-label, multicenter study (NCT02955069) exploring the antitumor activity of spartalizumab in previously treated WD NETs of intestinal, pancreatic, and thoracic origin and poorly differentiated GEP-NECs, 5 of the thoracic cohorts (6 TC and 24 AC) achieved a best response of PR^52^. Toripalimab, a humanized IgG4 antibody targeting the human PD-1 receptor, has been approved as a second-line therapy for metastatic melanoma. A phase Ib trial (NCT03167853) has investigated its efficacy in 40 patients with NENs that recurred or metastasized after first-line therapy, and reported an ORR of 20% and a median duration of response (mDOR) of 15.2 months^54^. Interestingly, the ORR in patients with PD-L1 expression ≥ 10% (50.0% *vs.* 10.7%, *P* = 0.019) or high TMB (75.0% *vs.* 16.1%, *P* = 0.03) suggests that toripalimab may be the most effective ICI therapy currently available for NENs, including G3 NET^54^. Although immunotherapy has been well tolerated as a treatment without any safety concerns, in contrast to previous trials in different tumor origins, its antitumor efficacy appears to be limited in patients with advanced and refractory NENs. Efficacy data for various ICIs in combination with other anticancer therapies have been explored in recent years.

### Advances in ICI-based combination regimens for neuroendocrine neoplasms

#### ICIs combined with chemotherapy

ICI combination chemotherapy has shown unsatisfactory results in NECs. A phase II two-arm clinical trial has investigated pembrolizumab and combination chemotherapy regimens (including irinotecan or paclitaxel) in patients with NECs with poor extrapulmonary differentiation who failed first-line chemotherapy (excluding MCC), but the ORR of the combination group was only 9%, and the mPFS and mOS were 2 and 4 months, respectively, thus suggesting that the efficacy of immune combination chemotherapy in poorly differentiated NECs is limited; however, the study sample size was small^58^. The phase II NICE-NEC trial was the first trial to assess the efficacy of nivolumab in combination with platinum-based doublet chemotherapy as a first-line treatment for GEP or unknown origin grade 3 NENs. Most patients had NECs (68.4%), the Ki67 positivity was > 55% (65.8%), the ORR for the combination treatment was 53%, the mPFS was 5.7 months, and the 12-month OS rate required further follow-up^55^. In the interim analysis of trial NCT03728361^71^, 12 patients (7 with GEP-NETs and 5 with pulmonary carcinoma) were treated with nalivolumab combined with temozolomide, with a PR of 25%, SD of 67%, and PD of 8%; however, the follow-up time was short, and follow-up data are expected. On the basis of the phase III IMpower133 trial^15^ and the CASPIAN trial^72^, the FDA approved ICI in combination with carboplatin and etoposide as a first-line therapy for patients with extensive-stage SCLCs. However, the phase III trial of ipilimumab in combination with chemotherapy for first-line treatment of extensive-stage-SCLCs indicated no evidence of prolonged OS, and the adverse events were comparable to those observed in the chemotherapy plus placebo group^73^. The CASPIAN phase III trial also assessed the efficacy of durvalumab plus platinum plus etoposide or in combination with tremelimumab, or chemotherapy alone. The results of the double immunization combined chemotherapy group are not yet available, the mDOR was 5.1 months, the ORR was 68% and 58%, and the mOS was 13.0 and 10.3 months (*P* = 0.0047), respectively, for the immunization plus chemotherapy group and the chemotherapy alone group, and no significant difference in safety data was observed^74^. In another phase II trial, paclitaxel in combination with pembrolizumab showed moderate activity as a second-line treatment after platinum-etoposide chemotherapy for SCLC, with an ORR of 23.1%, mDOR of 9.1 months, mPFS of 5.0 months, and mOS of 9.1 months. The most common grade 3–4 AEs were febrile neutropenia (7.7%), asthenia (7.7%), hyponatremia (7.7%), and type I diabetes (7.7%)^75^. The efficacy of ICIs in combination with other methods to enhance the immune response in SCLCs is currently being evaluated in multiple clinical trials, including ICIs combined with the agonistic monoclonal antibody utomilumab targeting CD137, INCAGN01876 targeting GITR, or INCAGN01949 targeting the CD134 costimulatory receptor; an antibody-drug conjugate targeting DDL3 (Rova-T); an inhibitor targeting multiple kinases, such as VEGF receptor, fibroblast growth factor receptor, and platelet-derived growth factor receptor; anlotinib; and the tubulin polymerization inhibitor plinabulin. Ongoing clinical trials of ICIs in combination with chemotherapy in NECs are detailed in **Table 1**.

**Table 1:** Ongoing clinical trials involving immunotherapy combinations for neuroendocrine tumors

Clinical trials	Phase	*N*	Line	Population	Regimen
NCT03980925	II	38	First-line	Metastatic SCLCs or poorly differentiated NECs	Nivolumab + etoposide + carboplatin
NCT03901378	II	/	First-line or second-line	Metastatic or unresectable recurrent LCNECs or GEP-NECs	Pembrolizumab + platinum + etoposide
NCT05058651	II/III	189	First-line	Extrapulmonary poorly differentiated, small cell NECs.	Atezolizumab + cisplatin/carboplatin + etoposide
NCT03591731	II	185	First-line or second-line	Metastatic or unresectable recurrent LCNEC or GEP-NECs	Nivolumab + ipilimumab
NCT04079712	II	30	≥Second-line	Advanced NECs, after the failure of at least one line of prior therapy.	Nivolumab + ipilimumab + cabozantinib
NCT04197310	II	35	First-line	Locally unresectable or metastatic nonpancreatic WD-NETs	Nivolumab + cabozantinib
NCT04400474	II	144	≥Second-line	Advanced and progressive neoplasms of the endocrine system	Cabozantinib + atezolizumab
NCT04579757	Ib/II	120	≥Second-line	Advanced solid tumors including low/moderate thoracogenic NETs	Surufatinib+ tislelizumab
NCT04525638	II	30	First-line or second-line	Advanced/metastatic, well-differentiated grade 3 NETs or NECs of the pancreas, gastrointestinal tract, lung and unknown primary site.	Nivolumab + 177Lu-DOTATATE
NCT02554812 (JAVELIN Medley)	Ib/II	398	First-line or second-line	Locally advanced or metastatic solid tumors including advanced/metastatic SCLCs	Utomilumab + avelumab
NCT03126110	I/II	145	≥Second-line	Advanced or metastatic malignancies including NETs	INCAGN01876 + nivolumab + ipilimumab
NCT03241173	I/II	52	≥Second-line	Advanced or metastatic malignancies including NETs	INCAGN01949 + nivolumab, ipilimumab, or both
NCT03026166	I/II	42	≥Second-line	SCLCs	Rova-T + nivolumab or nivolumab + ipilimumab
NCT04192682	II	40	≥Second-line	SCLCs	Anlotinib + sintilimab
NCT03575793	I/II	55	≥Second-line	Recurrent SCLCs	Plinabulin + nivolumab + ipilimumab
NCT03071406	II	50	≥Second-line	Metastatic MCCs	Nivolumab + ipilimumab ± SBRT
NCT03304639	II	100	First-line or second-line	Metastatic MCCs	Nivolumab + pembrolizumab or radiation therapy
NCT04261855	I/II	65	First-line	Metastatic MCCs	Avelumab + external beam radiation therapy or 177-Lu-DOTATATE
NCT02643303	I/II	58	Any number of prior systemic therapies	Metastatic MCCs	Tremelimumab + durvalumab + polyICLC

NETs, neuroendocrine tumors. NECs, neuroendocrine carcinomas. NENs, neuroendocrine neoplasms. WD, well differentiated. GEP, gastro-entero-pancreatic. SCLCs, small cell lung cancers. MCC, Merkel cell carcinoma. LCNEC, large cell neuroendocrine carcinoma. N, number of patients expected or actually enrolled.

#### ICIs in combination with anti-angiogenic therapy

A series of preclinical and clinical studies have shown that anti-angiogenic therapy and ICI therapy have mutually enhancing effects. On the one hand, anti-angiogenesis blocks negative immune signaling by increasing the ratio of anti/pro-tumor immune cells and decreasing multiple immune checkpoint expression. On the other hand, ICI treatment can restore the immune support microenvironment and promote vascular normalization. In addition, because vascular normalization enhances drug delivery benefits, lower doses of ICI may be applied, thereby decreasing the risk of adverse events^76^. Halperin et al.^77^ have reported that atezolizumab-based bevacizumab resulted in an ORR of 15%–20% and a PFS of 14.9–19.6 months for extrapancreatic and pancreatic grade G1 and G2 NETs, thus indicating synergistic activity of bevacizumab in converting immune “cold” tumors into “hot” tumors. In a phase I clinical study of toripalimab in combination with surufatinib, a small molecule tyrosine kinase inhibitor of VEGFR/fibroblast growth factor receptor/CSF-1R, in advanced solid tumors, the ORR was 44%, and the DCR was 87.5%^78^. Preliminary data from a phase II clinical study (NCT04169672) in a multicenter polyoma cohort have shown that 20 patients with evaluable NECs who received surufatinib in combination with toripalimab had an ORR and DCR of 20% and 70%, respectively, and an mPFS of 3.94 months; 33.3% experienced ≥ grade 3 TRAEs, and 28.6% and 19% discontinued the trial drug, surufatinib or toripalimab, respectively, because of TRAEs. Data on well-differentiated NETs have not been reported^79^. Several studies of anti-angiogenic agents in combination with immunotherapy are ongoing, and the specific trial contents are detailed in **Table 1**.

#### Combination therapy with dual ICIs

Treatments with dual checkpoint inhibitors using anti-PD-1/PD-L1 and anti-CTLA-4 antibodies have shown promising efficacy. The phase II CA209-538 clinical trial of an N plus I regimen in the treatment of NENs (NCT02923934)^80^ has demonstrated an overall ORR of 24%, DCR of 72%, and mPFS and mOS of 4.8 months and 14.8 months, respectively; the response rate of bronchial AC was 33%; the ORR was 43% and 33.3% in 7 patients with pNENs and 3 patients with GI-NENs, respectively; and all responders had high-grade disease. A phase II study (NCT04969887) evaluating N plus I in patients with immunotherapy-sensitive cancers (including NECs and G3 NETs) in CA209-538 is registered and is expected to be completed in October 2024. The phase II basket SWOG DART S1609 trial of N plus I in rare tumors (NCT02834013)^53^ included 33 patients with low-, intermediate-, and high-grade NETs and NECs, and has shown an ORR of 25% [1 complete response (44%) and 0 responses for high- and low-intermediate NENs, respectively], 6-month PFS of 31% (44% and 14% for high- and low-intermediate NENs, respectively), and mOS of 11 months, thus suggesting that high-grade NENs or NECs may benefit more from N plus I treatment. In terms of safety, 38% of patients experienced grade 3–4 irAEs, and 31.5% of patients discontinued treatment because of irAEs; therefore, immune doublet therapy requires attention to the management of irAEs^53^. Nonetheless, the SWOG DART S1609 trial was cited as class 2B evidence by the 2020 NCCN guidelines for neuroendocrine tumors, which recommend N plus I for the treatment of extrapulmonary non-pancreatic poorly differentiated NECs progressing after chemotherapy. Similarly, 3 included studies evaluated the efficacy of N plus I and consistently observed high ORRs of 24.1%–27.3%^50,65,80^. However, in the phase III Checkmate-451 trial, N plus I as a first-line maintenance therapy for SCLCs did not show an improvement in survival, and the incidence of all grade adverse effects was higher in the double immunization group (86%) than in the single agent treatment^81^. In addition, the prospective phase II DUNE trial explored the efficacy of ipilimumab in combination with durvalumab for NENs that failed standard therapy, including 4 cohorts: lung AC/TC (*n* = 27), G1 and G2 GI-NETs (*n* = 31), G1 and G2 pancreatic NETs (*n* = 32), and G3 GEP-NENs (*n* = 33, including 91% NECs)^82^. The DCR rate at 9 months was 7.4%, 32.3%, and 25% in the first 3 cohorts, and the OS rate at 9 months was 36.1% in the G3 GEP-NEN cohort; the irORR according to irRECIST criteria was 7.4%, 0%, 6.3%, and 9.1%, and the mPFS was 5.3 months, 8.0 months, 8.1 months, and 2.5 months in the 4 cohorts, respectively. The main grade 3 or higher AEs were hepatotoxicity (9.7%) and diarrhea (6.5%). Therefore, the combination of dual ICIs has limited efficacy and a relatively lower ORR for well-differentiated NETs, whereas it may be more worthy of further investigation for poorly differentiated NECs.

#### Other combination immune therapies

In addition to the above regimens, relevant prospective clinical trials of ICIs in combination with other therapies are currently being conducted in NET cohorts, such as spartalizumab in combination with LAG525 (NCT03365791)^83^, 177Lu-DOTA0-Tyr3-octreotate (Lu-177) in combination with nivolumab (NCT03325816)^84^, and pembrolizumab plus somatostatin receptor ligands (NCT03043664). Radiotherapy or peptide receptor radionuclide therapy before the initiation of ICI treatment and induction of inflammation at the tumor level, accompanied by an increase in TILs, may be another approach for exploration. IDO mediated immunosuppression is most prominent in patients with low tryptophan levels; therefore, these patients may be interesting candidates for ICI combined with IDO inhibitor therapy^85^. Owing to the abundance of TAMs, and their negative correlation with T cell infiltration in the TME of NETs, the combination of CD47 inhibitors with ICIs may also be an interesting option in future studies^86^. A phase II study (NCT02465957) is testing the advantage of activated NK-92 NK cell infusions in combination with ALT-803 (interleukin-15) in patients with advanced MCC. ALT-803 (NCT03228667) has also been administered in combination with a PD-1/PD-L1 inhibitor for as many as 16 cycles in patients with advanced solid tumors, including MCCs or SCLCs, that progressed after an initial response to PD-1/PD-L1 inhibition. Interferon-alpha (IFN-a) has been identified as a potential treatment modality for patients with NETs, and patients with metastatic or unresectable NETs including low proliferation rates are currently being recruited for a study evaluating whether this treatment regimen decreases the rate of circulating Tregs with a combination of cyclophosphamide and IFN-a (NCT02838342). In the future, IFN-a therapy in combination with ICIs should be investigated as a combination therapy. Survivin long peptide vaccine, an immune tumor vaccine against NETs, and dendritic cells loaded with autologous tumor homogenates have entered phase I and II clinical trials, respectively. In addition, epigenetic therapy and immunotherapy can be combined to effectively overcome cancer treatment conundrums^87^ and are worthy of exploration in NETs.

## Exploration of predictive biomarkers of ICI efficacy for neuroendocrine neoplasms

Because the benefits of immunotherapy are usually limited to a subset of patients, the research community has made great efforts to find predictive markers that can identify such patients^88^. A study in tumors that typically receive immunotherapy has identified biomarkers that may predict response to immunotherapy, including PD-L1 expression, TMB, neoantigen burden, and TILs. Evidence suggests that PD-L1 expression is associated with higher response rates and prolonged survival after anti-PD-1/PD-L1 therapy^89–91^. In a phase Ib trial of patients with NENs (Ki-67 ≥ 10%) treated with toripalimab, patients with PD-L1 expression ≥ 10% had a better ORR than patients with PD-L1 expression < 10% (50.0% *vs.* 10.7%, *P* = 0.019)^92^. However, the ORR in patients with pNETs with positive PD-L1 expression in the Keynote-28 study was low, at 6.3%^50^; all 4 patients with GEP-NETs who achieved PR in the Keynote-158 study had negative PD-L1 expression^51^; no differences in DCR, PFS, or OS were observed between the PD-L1-negative and PD-L1-positive arms with G3 NENs in the combined analysis of the 2 prospective, non-randomized trials^16^. In fact, PD-L1-negative tumors also respond well to anti-PD-1/PD-L1 therapy^93^. Therefore, PD-L1 must be combined with other predictive biomarkers to better predict the populations that may benefit from immunotherapy. Large clinical and genomic data sets have shown that high TMB is associated with prolonged survival in patients treated with ICI for various cancer types^94^. Although pembrolizumab has been approved by the FDA for patients with TMB ≥ 10 mut/Mb, on the basis of the results of the Keynote-158 trial, regardless of the primary tumor, significant differences exist in TMB among tumor types, and the optimal TMB threshold for each histology is controversial^95^. In addition, high TMB may be associated with a higher proportion of immunogenic cancer-specific “neoantigen” burden, but these “neoantigen” proteins must be effectively presented and expressed^96^. In May 2017, the FDA approved pembrolizumab for patients with unresectable or metastatic microsatellite instability (MSI)-high or mismatch repair-deficient (dMMR) solid tumors progressing after prior therapy^97,98^. However, analysis of 2 studies including 89 patients with small intestinal NETs and 35 patients with pNETs has suggested that in NETs, DNA dMMR is rare, and tumors have microsatellite instability^99,100^. A study investigating NECs (*n* = 53) and mixed adenoneuroendocrine carcinomas (*n* = 36) has indicated that 12.4% of patients with these carcinomas had MSI^101^.

Immune cell infiltration in the TME is one of the most essential features for generating an appropriate antitumor immune response. An observational study of 87 patients with NETs has found that in primary moderate NETs, intensive CD3^+^ T cell infiltration was associated with a relapse-free survival of 128 months, whereas patients with low intratumoral T cell levels had a recurrence free survival of only 61 months^102^. In the same study, an analysis of 39 patients with NETs with liver metastases showed that the degree of infiltration of CD3^+^, CD4^+^, and CD8^+^ did not predict OS, whereas low levels of infiltrating Tregs predicted prolonged OS^102^. In addition, chronic inflammation can overstimulate neuroendocrine cells, thus leading to hyperplasia and neoplastic transformation. The neutrophil-lymphocyte ratio and platelet-lymphocyte ratios are simple and effective biomarkers available for patients with advanced cancer including NENs, and their prognostic roles have been confirmed in 15 and 4 studies, respectively; however, the thresholds for both ratios remain undefined^103^. Finally, the specific composition of the gut microbiome has been shown to influence antitumor immune responses, but no data are available on the gut microbiomes of patients with NETs and NECs treated with ICIs. An in-depth study of the immune microenvironment and the exploration of novel markers are crucial tasks, but the predictive efficiency of molecular markers confirmed by current studies remains unsatisfactory, and the study sample sizes have been small. More predictive immunotherapeutic markers must be explored to identify so-called “hot” tumor lesions and guide immunotherapy.

## Summary and future prospects

NENs are a rare, complex and highly heterogeneous class of tumors with poor prognosis and limited treatment options for patients after failure of first-line therapy. Immunotherapy may be a powerful means of improving treatment efficacy in such patients, but the optimal strategy remains to be determined. The response rate to ICI monotherapy is low, the disease control time is short, and treatment may be effective for only a portion of the population. The low TMB and often “cold” immune microenvironment suggest that combination therapy may be used to overcome the intrinsic resistance of NENs to immunotherapy, including immune combination chemotherapy or somatostatin analogues and anti-angiogenic drugs, double ICI combinations, or simultaneous combination of anti-angiogenic drugs, to improve patient outcomes. In addition, accumulating experimental and clinical evidence supports that the interaction between neuroendocrine and immune systems is essential to maintaining homeostasis, and assessment of this broad neuroendocrine-immune correlation is essential for NENs. Future efforts should focus on finding the best way to incorporate immunotherapy into NEN treatment, including defining the most appropriate treatment context, combination, and treatment sequence. In addition, accurate molecular typing and immune monitoring are the only way to find markers for combined prediction of therapeutic effects and adverse reactions. However, the overall predictive efficiency of known biomarkers, such as high TMB, PD-L1 and MSI high/dMMR, is poor at present. Gene mutation types, T cell regulation-associated factors, and pathways of NENs involved in immunotherapy must be identified, and a combination of multidimensional, stereoscopic, and dynamic markers must be used to improve the predictive efficacy of markers; reveal the molecular mechanism of PD-1 antibody therapy and the causes of drug resistance; and guide the clinical practice of NEN immunotherapy in the future.
